# Dual Dimensionality Reduction Reveals Independent Encoding of Motor Features in a Muscle Synergy for Insect Flight Control

**DOI:** 10.1371/journal.pcbi.1004168

**Published:** 2015-04-28

**Authors:** Simon Sponberg, Thomas L. Daniel, Adrienne L. Fairhall

**Affiliations:** 1 Department of Biology, Univ. of Washington, Seattle, Washington, United States of America; 2 Department of Physiology & Biophysics, Univ. of Washington, Seattle, Washington, United States of America; 3 Institute for Neuroengineering, Univ. of Washington, Seattle, Washington, United States of America; 4 Program in Neuroscience, Univ. of Washington, Seattle, Washington, United States of America; Indiana University, United States of America

## Abstract

What are the features of movement encoded by changing motor commands? Do motor commands encode movement independently or can they be represented in a reduced set of signals (i.e. synergies)? Motor encoding poses a computational and practical challenge because many muscles typically drive movement, and simultaneous electrophysiology recordings of all motor commands are typically not available. Moreover, during a single locomotor period (a stride or wingstroke) the variation in movement may have high dimensionality, even if only a few discrete signals activate the muscles. Here, we apply the method of partial least squares (PLS) to extract the encoded features of movement based on the cross-covariance of motor signals and movement. PLS simultaneously decomposes both datasets and identifies only the variation in movement that relates to the specific muscles of interest. We use this approach to explore how the main downstroke flight muscles of an insect, the hawkmoth *Manduca sexta*, encode torque during yaw turns. We simultaneously record muscle activity and turning torque in tethered flying moths experiencing wide-field visual stimuli. We ask whether this pair of muscles acts as a muscle synergy (a single linear combination of activity) consistent with their hypothesized function of producing a left-right power differential. Alternatively, each muscle might individually encode variation in movement. We show that PLS feature analysis produces an efficient reduction of dimensionality in torque variation within a wingstroke. At first, the two muscles appear to behave as a synergy when we consider only their wingstroke-averaged torque. However, when we consider the PLS features, the muscles reveal independent encoding of torque. Using these features we can predictably reconstruct the variation in torque corresponding to changes in muscle activation. PLS-based feature analysis provides a general two-sided dimensionality reduction that reveals encoding in high dimensional sensory or motor transformations.

## Author Summary

Understanding movement control is challenging because the brains of nearly all animals send motor command signals to many muscles, and these signals produce complex movements. In studying animal movement, one cannot always record all the motor commands an animal uses or know all the ways in which movement varies in response. A combined approach is necessary to find the relevant patterns: the changes in movement that correspond to changes in the recorded motor commands. Techniques exist to identify simple patterns in either the motor commands or the movements, but in this paper we develop an approach that identifies patterns in both simultaneously. We use this technique to understand how agile flying insects control aerial turns. The two main downstroke muscles of moths are thought to produce turns by creating a power difference between the left and right wings. The moth’s brain may only need to specify the difference in activation between the two muscles. We discover that moth’s brain actually has independent control over each muscle, and this separate control increases the moth’s ability to adjust turning within a single wingstroke. Our computational approach reveals sophisticated patterns of movement processing even in the small nervous systems of insects.

## Introduction

Control of animal movement is accomplished through the coordinated action of many parallel motor signals activating many muscles. To understand how motor spikes are transformed into action requires knowledge of how movement is encoded in these patterns of neuromuscular activation. Unfortunately, we cannot reliably predict the movement resulting from a particular motor signal simply from a muscle’s anatomy and static function (e.g. an “extensor”) [1–4]. The same pattern of activation to the same muscle, but in different dynamic contexts can even produce turning torques in opposite directions [2]. Moreover, both motor signals (the inputs to the motor transform) and movements (the outputs) are typically high dimensional and we may not be able to record all relevant motor signals electrophysiologically. Understanding how muscles work together to encode movement is therefore a computational challenge of both 1) dimensionality and 2) incomplete representation.

High dimensionality is ubiquitous in both sensory and motor transformations. Dimensionality reduction techniques used in sensory neuroscience are typically one-sided, meaning that a high dimensional stimulus is reduced to describe variation in a single spiking neuron [5]. Similar use of one-sided dimensionality reduction in the motor transformation identifies patterns in high dimensional representations of movement encoded in the activity of individual muscles e.g. [6]. Conversely, the dimensionality of the neural signals can be high, as is the case for many central brain recordings, but in many experimental designs the representation of motor output is restricted to few dimensions, or even discrete states, e.g. [7]. Developing techniques that represent high dimensional movement encoded in high dimensional neural signals remains challenging [8], yet is ever more pressing as such datasets become the norm.

The second challenge for motor encoding is one of incompleteness. Muscle synergies, patterns of variation in activation across multiple muscles, are hypothesized to reduce the dimensionality of the motor commands and provide high level encoding of movement features [9,10]. However, not all variation in muscle activation might affect movement dynamics (and vice versa). If we consider all variation in a high dimensional description of movement as potentially relevant, we are likely to include variation that is not encoded by the muscles we are able to record from. This challenge exists for sensory encoding as well. Only certain features of a complex stimulus may be encoded by the neurons under consideration. Spike triggering is one way to extract only relevant variation. This method conditions the stimulus on the spiking of an individual neuron (or muscle) thereby limiting the reduced stimulus description to what is encoded in that spike. However, we can only align to a single neuron or discrete patterns of spiking across many neurons [5,6]. Relating activation of multiple muscles to a rich description of movement demands a reliable way to 1) reduce dimensionality when both input and output have multiple dimensions and 2) extract only the changes in movement that covary with the subset of muscles recorded.

Here, we develop a feature encoding analysis based on the partial least squares (PLS) method of two-sided dimensionality reduction [11,12]. By two-sided, we mean that PLS uses variation in both input and output to reduce dimensionality, addressing the two challenges above. Using this approach, we analyze the movement encoded in the flight muscles of the hawkmoth, *Manduca sexta*, to test a muscle synergy hypothesis for flight muscle coordination [13,14].

The term “muscle synergy” has many meanings [9,10,15–20], but it is usually represented as a set of muscles that act in a fixed proportion, or in proportions undergoing a fixed time-varying pattern [10,20]. In their most general sense, muscle synergies are linear combinations of variables describing muscle activation that capture variation with fewer dimensions than the complete set of variables [9]. To avoid confusion, this use of muscle synergy differs from “information synergy” where two signals jointly provide more information than the sum of their individual contributions [21]. It is also different from the terminology of synergistic (vs. antagonistic) muscles, which refers to muscles acting on the same joint to produce movement in the same (vs. opposite) direction.

**Fig 1:**
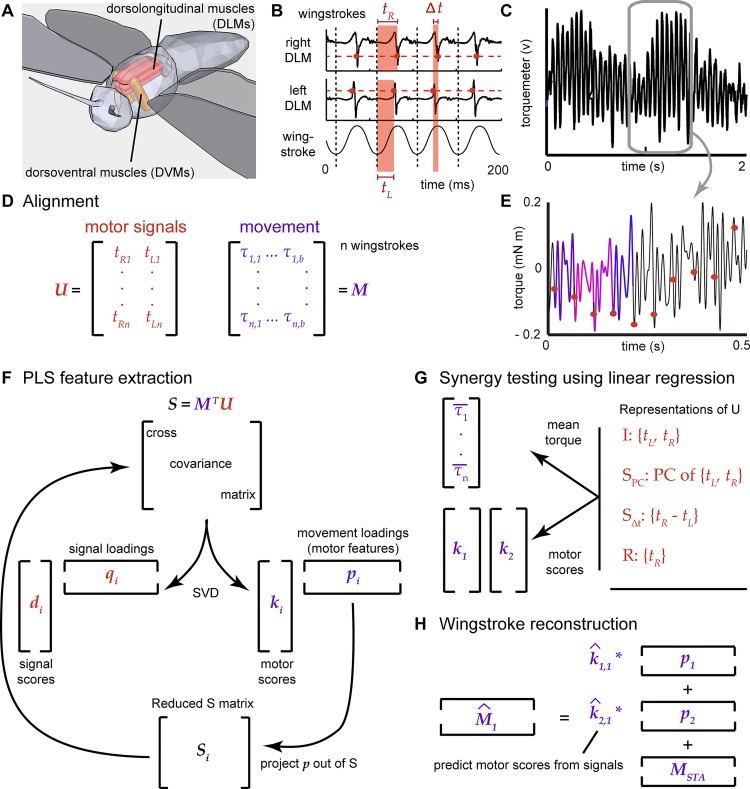
Encoding of torque in muscle activation. Each dorsolongitudinal muscle (DLM) is composed of five separately innervated subunits. The subunits fire simultaneously with a single muscle action potential acting as a combined motor unit [23]. Contraction of the DLMs produces the downstroke through deformation of the thorax, which is restored by the upstroke dorsoventral muscles (DVMs) (A—only main flight muscle shown; sub-figure derived from [14]). There are three major groups of steering muscles (axillary, basalar, and subalar) which are not shown for clarity, but lie just lateral to the DLMs and DVMs, near the wing hinge (see [24]). DLM recordings during tethered flight (B) are parameterized by the spike timing variables (*t*_*L*_, *t*_*R*_). Activation varies in the timing of the spike, not the number of spikes. Changes in timing can produce variation in yaw torque (C). In PLS feature analysis (D-H), we relate these motor signals (the ***U*** matrix) to the turning torque (the ***M*** matrix) (D). The raw torque signal is calibrated to extract the within-stroke torque (E). We then spike-trigger (dots in e; each of the first four extracted torque chunks is colored) and align the torque (D), find a reduced dimensional feature space via the partial least squares (PLS) method (F), test if synergy models can account for this feature space (G), and reconstruct the torque waveform from the timing variables (H).

A variety of reasons have been posed for the existence of synergies, most notably for simplifying control by reducing the number of independent motor signals an animal’s brain must control [10]. In invertebrates some synergies exist simply due to anatomy because individual motor neurons can innervate multiple muscles (e.g. [22]). Even when innervation is separate, motor units can fire in very tight synchrony, acting like a simple synergy. In *Manduca*, each of the main downstroke muscles (dorsolongitudinal muscles or DLMs) has five discrete subunits (Fig 1A), each innervated by a separate motor neuron (one per subunit) [13,23]. However, the whole muscle fires as one combined motor unit because the timing of the motor neurons is very precisely synchronized [23]. In fact, each DLM is driven by only a single muscle potential during each wingstroke (Fig 1B; [13,14]). Activation of the DLMs therefore varies only in the timing of the spike rather than magnitude (e.g. number of spikes). During straight flight the timing of the left and right DLM are also very precisely synchronized (spikes occur within < 1 ms of each other), but during turning the timing is modulated over an ~8 ms window [14].

The fact that the DLMs have any potential for control has only recently been appreciated [14,25] because this subtle timing modulation occurs over such a narrow range. Nonetheless, such small shifts produce large changes in mechanical power output [14,26]. Prior to this discovery, control of the wings was thought to be the exclusive domain of the small steering muscles that trim and tension the wingstroke [13].

The question we pose here is whether the two DLMs encode independent aspects of turning torque production or if they act as a synergy. Three reasons why these muscles could act as a synergy are: 1) They are activated at the midpoint of a steep but monotonic region of the power-phase curve, meaning that small changes in timing produce large, but nearly linear, changes in power output [14,26]. Therefore the timing difference between the left and right muscle’s spike translates into a power differential [14]. 2) Variation in the timing of activation is also highly correlated between the DLMs, meaning that little independent variation exists that could encode movement separately in each muscle [14]. 3) The two muscles both attach to the same large exoskeletal plate that deforms to drive wing motions, resulting in mechanical coupling of their action [24]. As we will see, the synergy hypothesis is supported when we consider wingstroke-averaged turning torque, but fails to describe the variation in motor features captured by the PLS analysis.

The tractability of analyzing invertebrate motor commands combined with the ability to record turning torque affords an opportunity to test hypotheses about muscle synergies with spike-level resolution. The coordination of the moth’s downstroke muscles is a very simple synergy hypothesis: that one variable describing the combination of these two muscles’ activities has as much predictive power as considering the two muscles independently. *Manduca* flight muscle is also a good system for assessing the PLS approach because the timing of activation of multiple muscles translates into a continuously varying, high dimensional pattern of torque throughout the wingstroke. Our goal is to identify the few relevant dimensions of torque that are encoded by changes in the DLMs and then to use these to assess the synergy hypothesis. Even though we focus on a specific hypothesis about an insect’s flight motor program, we use this system to show how PLS feature analysis may be generally applied to produce a data-driven decomposition of two simultaneously measured datasets.

## Materials and Methods

### Experimental data

*Manduca sexta* is a large crepuscular hawkmoth capable of agile, maneuverable flight. It demonstrates a strong visual tracking (optomotor) response to oscillating wide-field optical patterns [27]. This behavior is ecologically relevant because moths must hover and feed while visually tracking flowers’ movement [28,29]. Flight in hawkmoths is powered by a left-right pair of DLMs and a pair of upstroke muscles (the dorsoventral muscles or DVMs; Fig 1A) [24]. However, a suite of several smaller steering muscles further trim or tension the movement of the wings, and contribute to the control of turning [13,30]. It is therefore still challenging to isolate the turning dynamics encoded in the DLMs’ activation alone.

We flew seven moths (mixed sex) under open-loop, visually driven (i.e. optomotor) flight conditions that produced left and right turning behaviors. The data used here are from the same moths used previously and under conditions elaborated on in [14]. In brief, bipolar tungsten electrodes inserted through the scutum on the dorsal surface of each moth recorded from the DLMs (Fig 1B). We tethered moths to a custom optical torque-meter that produced an output voltage dependent on the yaw (left-right) turning torque of the animal (Fig 1C) [14]. Following at least a minute of warm-up shivering, moths produced full-amplitude wingstrokes spontaneously or when we elicited flight with a light touch to the neck region. The visual stimulus consisted of a sinusoidal grating of light and dark bars with a spatial frequency of 0.05 cycles degree^-1^, oscillating sinusoidally at 1 Hz. Because wingstroke frequency is much faster (~25 Hz), this slow variation in optic flow magnitude produced individual wingstrokes spanning a wide range of average yaw torque. We recorded electromyograms (EMGs) from the left and right DLMs and detected spikes using simple threshold crossing. Invertebrate EMGs usually afford resolution of individual muscle potentials, or spikes, whereas vertebrate recordings typically record from many motor units simultaneously, obscuring individual spikes [9,10]. Trials were analyzed further only if the right DLM’s average spike rate exceeded 18 spikes sec^-1^, corresponding to the lowest flight flapping frequency.

To extract the torque within each wingstroke, we had to decouple the internal dynamics of the torquemeter from the forces applied by the animal. We modeled the torquemeter as a forced, damped rotational oscillator.

$$I\ddot{\phi }+C\dot{\phi }+\kappa \phi =\tau (t).$$ (1)

where *ϕ* is the angle of rotation, *I* is the moment of inertia, *C* is the torsional damping coefficient, and *κ* is the torsional stiffness. We fit the spring, damping, and inertial parameters using a series of calibration trials following [31].

### Analytical approach & synergies

Our analytical goal was to relate a measured set of motor signals to the resulting movement, extract the relevant dimensions of variation in movement, and use this set of motor features to test the synergy hypothesis. During periodic movement, the motor signals form a matrix ***U*** of *k* timing (or phase) variables {*u_1_*, …, *u_k_*}, characterizing neural or muscular action potentials (hereafter “spikes”) for each of *N* periods of movement (e.g. wingstrokes). During rhythmic movement with a characteristic period *T*, the motor output matrix is an *N* x *b* matrix, *M*, where *b* is the number of samples of a set of movement variables over *T*.

Since the DLMs each only receive a single spike of activation per wingstroke, we defined the *N* x 2, ***U*** signal matrix using the time of the left (*t_L_*) and the right (*t_R_*) muscle’s spikes relative to the zero phase onset of each wingstroke (Fig 1B). These variables were each centered and scaled by their variances. The N x 500, ***M*** movement matrix was composed of 50 ms (500 samples) long waveforms, {*τ*_1_ .… *τ*_500_}, based on a typical tethered wingbeat frequency of 20 Hz and a 10 kHz sampling of torque. The ensemble of wingstrokes were centered and scaled by their overall variance, *s*. Our approach to extracting motor features follows four steps (Fig 1D–1H): 1) *Alignment* of the torque waveforms, 2) *dimensionality reduction* of the movement matrix, ***M***, to extract a reduced basis, termed motor features, 3) *synergy testing* and 4) *reconstruction* of the torque waveforms from the motor signals, ***U***.

By comparing how well torque was encoded by different combinations of the motor signals, ***U***, we asked if these signals act as a synergy or independently encode information about movement. In the independence model, the activation of each muscle {*t_L_*, *t_R_*} contributes significantly to predicting torque, and these two variables (the two columns of ***U***) cannot be reduced to a single variable.

We pose two synergy models, constructed as different linear combinations of the motor signals. The first is based on an *a priori* physiological hypothesis that the timing difference Δ*t* between the muscles’ spikes could translate directly into a power differential between the muscles [14,25]. We refer to this as the differential synergy model. The second synergy model is entirely data-driven: we extract the first principal component (*t_PCA_*—the PCA synergy model) of the two motor variables {*t_L_*, *t_R_*}. This closely mirrors the existing methods of muscle synergy calculation in the literature [9]. However, we use PCA instead of the sign-dependent nonnegative matrix factorization (NMF) technique [32] because timing can shift positively or negatively. We call this the empirical synergy model. Finally, we test a redundancy model, in which the variation in torque is equally well explained by only one muscle’s activation.

In summary, we use the full set of motor signals, ***U***, and torque samples, ***M***, to identify all the variation in movement that cross-correlates with changes in any ***U*** variable (Fig 1F). Using this same feature basis, we then test whether either of the synergy models can fully explain the variation in the feature basis or if the independence model (full ***U***) is needed (Fig 1G). This avoids circularity because we identify the torque features contingent on ***U***, but all the synergy and independence models are subsets or combinations of ***U***. We test the different synergy, independence, and redundancy models by how well they explain variation in the projection of torque onto the feature basis (the PLS “scores”) and also how well the competing models perform in reconstruction of the entire torque waveform (Fig 1H).

### Alignment of torque waveforms

**Fig 2:**
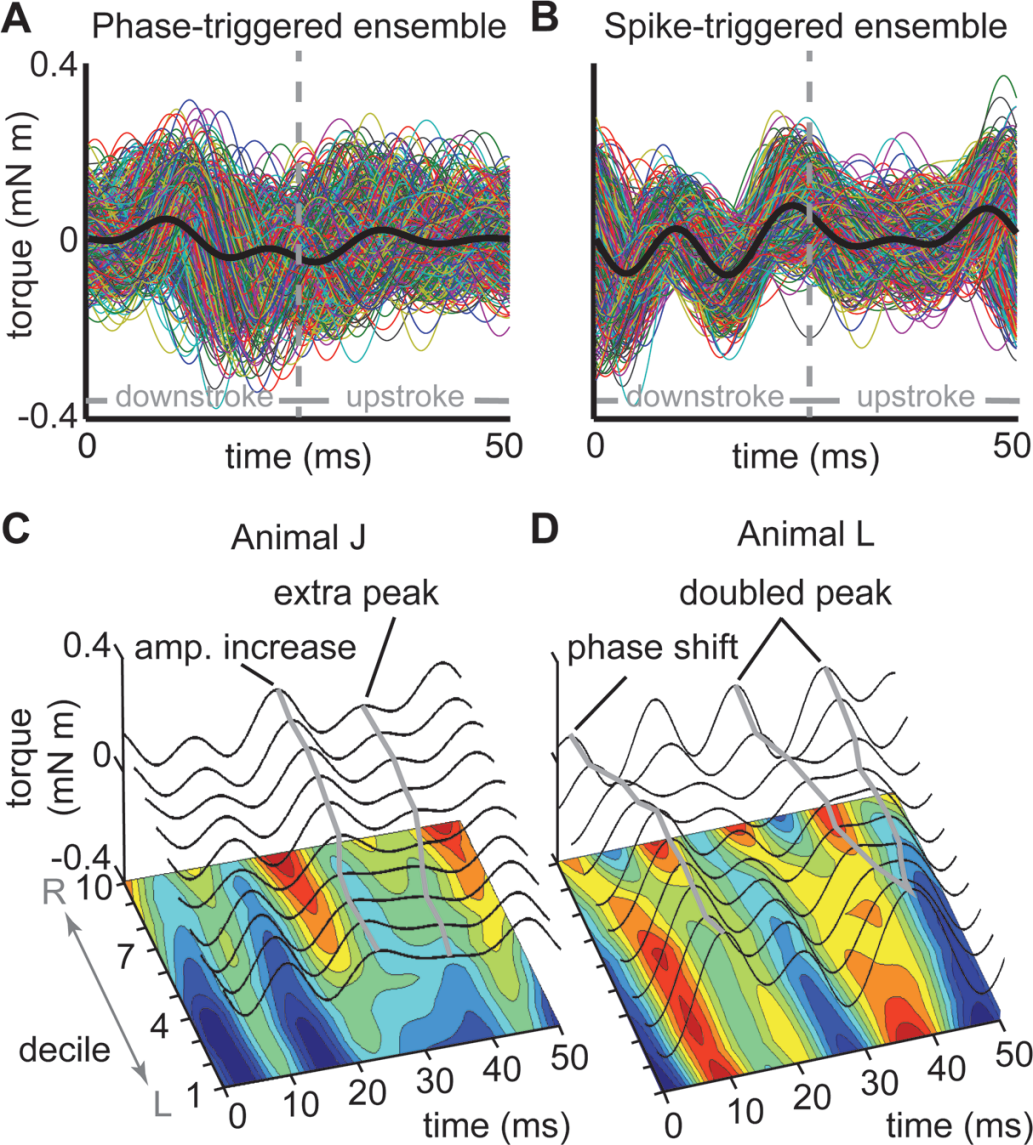
Motor-spike-triggered ensembles and turning deciles. We aligned the torque waveforms to either the zero phase crossing (A) or the timing of the right DLM’s spike (B). We then divided the data into deciles ordered by average torque. The decile averages (C, D) and the interpolated contour surfaces (below) from two animals (“J” and “L”) show the range of torque variation within wingstrokes. Deciles are ordered from the greatest leftward (”L”) to rightward (”R”) torque. Grey transects highlight distinctive features mentioned in the text. Because wingstrokes are triggered on the right muscle’s spikes, we do not expect these patterns to be left-right symmetric.

We aligned the torque signal from each wingstroke in two different ways. We first produced an alignment that did not depend on the spikes directly. We computed the Hilbert transform of the torque signal, and filtered around the dominant 20 Hz wingstroke frequency (3–35 Hz bandpass, 8^th^ order Type II Chebychev). The Hilbert transform returns a periodic function whose value estimates the phase of the original time series data and has been used for both gait [33] and rat whisking analyses [34]. After transforming the raw voltage signal (Fig 1C) into actual torque (Fig 1E), each torque segment was aligned to the zero phase crossing of each wingstroke (“phase-triggered”). As an alternative, we also aligned the torque to the timing of the right DLM’s action potential (“spike-triggered”; Fig 1E). In both alignments, the resulting wingstrokes were assembled into the movement matrix, ***M*** (Fig 2A and 2B).

### Dimensionality reduction & feature extraction

We applied two dimensionality reduction methods to the two different waveform alignments to determine which best captured the variations in torque that correspond to the motor timing variables in ***U***. We first applied standard principal component analysis (PCA) by computing the eigendecomposition of the covariance matrix of ***M*** alone for both the phase- and spike-triggered ensembles. Note that this is different from the PCA used on the motor signals, ***U***, to create the empirical synergy model. We compared the one-sided PCA analysis of ***M*** with a two-sided, cross-covariance decomposition based on the partial least squares (PLS) method (Fig 1F). We explored how effectively these reduced descriptions of the torque output (termed motor features) captured variation in the motor signals {*t_L_*, *t_R_*} via standard regression of the features onto the signals.

### PLS

Partial least squares regression, hereafter PLS, extracts features from one dataset of predictor variables, here the animal’s movement ***M***, to maximize each successive feature’s cross-covariance with an arbitrarily large set of predicted variables, here the motor signals ***U*** (Table 1) [11,12,35]. PLS regression is one of several methods based on Wold’s original Projection onto Latent Structures [11], which are used widely in chemometrics [35]. PLS regression uses an iterative approach that greedily extracts features in one dataset (here ***M***) that maximally predict the remaining variance in another dataset (here ***U***). The set of features identified from PLS therefore are not guaranteed to maximize a global statistical property of the data [36], although each subsequent feature is optimal for that iteration. This approach has been shown to have good predictability in empirical datasets ranging from neural imaging analyses [37], ecology [38], geometric morphometrics [39,40], and paleontology [41]. We implement the faster Statistically-Inspired Modification of PLS (SIMPLS) developed by de Jong [35] rather than the original Non-Linear Iterative PLS (NIPALS) [11] that requires iterative optimization steps.

**Table 1:** PLS regression summary.

motor signals (neuromuscular)		motor output (torque)	
	Predictor var. →		***M***
***U***	← Explained var.		
***C***, ***c***_1_	Weights (SVs)		***R***, ***r***_1_
***Q*, *q***_*1*_	Loadings (features)		***P***, ***p***_1_
***D*, *d***_*1*_	Scores (coefficients)		***K***, ***k***_1_

Because we extract features of the motor output that maximize their ability to reconstruct the motor signals, the direction of the PLS regression is from ***M*** to ***U***. This also allows the number of relevant motor output features to exceed the rank of ***U***. The *weights* are the left and right singular values (SVs) from an SVD of the cross covariance matrix of ***M*** and ***U***. Projecting these into ***M*** and ***U*** gives the *loadings*, which are the features and the coefficients of these projections are the *scores*. The SIMPLS algorithm, applied to ***U*** and ***M***, has the following steps:

1. Construct the cross-covariance matrix:
  $$S_{0}=M^{T}U.$$ (2)
2. Compute ***r***_1_ and ***c***_1_, the leading right and left singular value decompositions of ***S*** *_0_*. These are the “weight” vectors of ***M*** and ***U*** respectively.
3. From these weight vectors, the motor output “scores” vector ***k***_*1*_ is computed as:
  $$k_{1}=Mr_{1}.$$ (3)
  These scores are then normalized:
  $$k_{1}=\frac{k_{1}}{\left\|k_{1}\right\|}.$$ (4)
4. The scores represent the amount of the leading feature represented in each wingstroke. The motor output “loadings” vector ***p*** *_1_*, defined as:
  $$p_{1}=M^{T}k_{1},$$ (5)
  is the first motor feature itself and is analogous to a principal component or eigenvector.
5. The loadings (***q*** *_1_*) and scores (***d*** *_1_*) of ***U*** are calculated from ***k*** *_1_*, making the analysis asymmetric.
  $$q_{1}=U^{T}k_{1},$$ (6)
  $$d_{1}=Uq_{1}.$$ (7)
6. This leading feature is projected out of the current ***S*** *_0_* matrix, which is “deflated” to obtain a new cross-covariance matrix ***S*** *_1_*:
  $$S_{1}=S_{0}-v_{1}v_{1}{}^{T}S_{0},$$ (8)
  $$v_{1}=\frac{p_{1}}{\left\|p_{1}\right\|}.$$ (9)
  This is the same as first estimating the regression coefficient *b_1_*
  $$b_{1}=k_{1}{}^{T}u_{1},$$ (10)
  and then projecting out the newly obtained feature from ***M*** and ***U***:
  $$M_{1}=M-k_{1}p_{1}{}^{T}$$ (11)
  and
  $$U_{1}=U-b_{1}k_{1}c_{1}{}^{T}.$$ (12)
  S_1_ can then be reconstructed from the residual ***M***_1_ and ***U***_1_ matrices:
  $$S_{1}=M_{1}{}^{T}U_{1}.$$ (13)
7. Steps 2–5 are iterated to pull out the next set of scores, weights, and loadings {***k*** *_i_*, ***d*** *_i_*, ***r*** *_i_*, ***c*** *_i_*, ***p*** *_i_*, ***q*** *_i_*, ***v*** *_i_*}. However, each new ***v*** *_i_* is made orthogonal to all prior (normalized from Equation 9) features, ***V*** = {***v*** *_1_*, …, ***v*** *_i-1_*}, using a modified Gram-Schmidt process:
  $$v_{i}=v_{i}-V\;V^{T}v_{i}.$$ (14)
  In addition, new ***U*** scores (***d*** *_i_*) are made orthogonal to all previous ***M*** scores, ***K*** = {***k*** *_1_*, …, ***k*** *_i-1_*}, so that only the independent variation explained in ***U*** is considered for each feature beyond the first:
  $$d_{i}=d_{i}-K\;K^{T}d_{i}.$$ (15)
8. The final feature basis of ***M*** is composed of the *n* features ***P*** = {***p*** *_1_*, …, ***p*** *_n_*} and their scores: ***K*** = {***k*** *_1_*, …, ***k*** *_n_*} that explain significant variation in one or more of the motor commands in ***U***.

In using cross-covariance to isolate relevant variation, PLS-based methods are similar to canonical correlation analysis (CCA) [42]. However, CCA is symmetric, uses only a single cross-covariance decomposition, and only extracts a number of features up to the smaller of the rank of the inputs or the rank of the outputs (here only two). In contrast, PLS is a greedy, iterative algorithm that captures the unique contributions of successive features in describing the motor signals while preserving the asymmetry through a regression step [12]. It can therefore produce a number of features up to the number of variables describing the movement, given by the dimensionality of ***M***.

### Synergy model testing

We compared the synergy and independence models first by how well the motor signals could predict the magnitude of each feature (its score) and the mean torque produced over a wingstroke (Fig 1G). We quantify model performance using the predicted residual sum of squares (PRESS). This form of leave-one-out cross-validation sequentially withholds each individual wingstroke from the analysis and then predicts the withheld wingstroke. If the two-variable independence model has greater predictive power than the one-variable synergy or redundancy models, then each muscle contributes significantly to the decoding of torque. If the independence model does not significantly improve PRESS, then at least one of the reduced models are favored. Note that the independence model cannot explain less variance than the synergy or redundancy models because these two alternatives are subsets of the independence model with one fewer free parameters. We used ANOVAs and paired tests to compare across models using each animal as a separate observation for model testing.

### Torque waveform reconstruction

In addition to comparing how well motor signals encoded the features, we also tested how well we could reconstruct the wingstroke torques from the features (Fig 1H). The torque waveforms, ***M***′, can be approximated from PLS features as a sum of the average motor output (the spike-triggered average—***M*** *_STA_*) and the features weighted by their scores:

$$M\prime \;=M_{STA}+\;sKP^{T}.$$ (16)

The scaling factor, *s*, corrects for the original centering and scaling of the ***M*** matrix. The final score and loading vectors for the motor signal ***U*** matrix, ***D*** = {***d*** *_1_*, …, ***d*** *_n_*} and ***Q*** = {***q*** *_1_*, …, ***q*** *_n_*} define the dimensions of ***U*** maximally predicted in a least squares sense by each feature, ***p*** ***_i_***. This approach is similar to reverse reconstruction of the stimulus given a set of spikes [5].

We first used the STA of all torque waveforms as a comparison for reconstruction (only the first term of Eq. 16). To demonstrate the efficacy of wingstroke-averaged methods, we next considered the STA with just the average torque over the whole wingstroke, < *τ* >, reconstructed from the synergy and independence models and added as a constant offset (the mean torque models). This is the same as assuming that only wingstroke-to-wingstroke changes are encoded in the motor signals.

We then reconstructed the torque using Equation 15, which includes the STA and the first two motor features:

$$M\prime \;=M_{STA}+\;sk_{1}p_{1}{}^{T}+sk_{2}p_{2}{}^{T}.$$ (17)

These reconstructions are the reduced-dimension representations of each measured wingstroke derived from the feature analysis. Finally, we reconstructed these waveforms from the motor signals themselves by predicting the features’ scores (***k***_*1*_ and ***k***_*2*_ in Eq. 17) from the motor signals. These reconstructions were based on using the independence, redundancy, differential synergy, and empirical synergy representations of ***U*** to generate the ***k***’s (via the regression equations).

As our main metric of reconstruction performance we used the RMS power of the residual (error) torque waveform normalized to RMS of the actual waveform. We also considered the residual, or unexplained, variance in the model, calculated as 1—*r* ^2^, where *r* is the correlation coefficient. RMSE is sensitive to small, but systematic deviations (e.g. offsets) whereas unexplained variance is sensitive to small phase shifts. Throughout the analyses we used repeated measures ANOVAs with each animal contributing an observation for each decile of turning and for each model (i.e. model and decile were each factors). Since we were primarily interested in comparing to the best model (the actual two feature waveform or the independence model), we used Hsu’s “multiple comparisons to the best” (MCB) test [43]rather than a Tukey comparison of all pairs. In paired comparisons we use paired t-tests. We confirmed that non-parametric tests (Kruskal-Wallis and pairwise Wilcoxon tests) did not affect our conclusions. Statistical tests were performed in Matlab (Mathworks, Natick, MA, USA) with Hsu’s MCB tests performed in JMP (SAS Institute, Cary, NC, USA).

To test the predictive power of the reconstructions, we cross-validated the feature analysis using 70% of each decile of the data as a training set to predict the remaining 30%. Cross-validation was repeated one thousand times. We also reconstructed each individual wingstroke’s torque, rather than the decile averages. In this case, the maximum reconstruction performance is likely to be limited because the motor commands from the main flight muscles should only predict a portion of the overall variation in the wingstroke. However, the ability of these motor commands to predict the scores (***k***_***i***_) of each PLS feature (***p***_***i***_) should remain high because these include only variation in torque corresponding to flight muscle variation.

## Results

### Aligned wingstrokes reveal patterns of torque variation

We first determined whether the torque waveforms varied in more than just their mean. After alignment via phase- or spike-triggering, we separated the resulting ensembles of wingstrokes into deciles ordered from left to right turns by mean torque. We found substantial variation, confirming our ability to visually induce a range of motor outputs (Fig 2C and 2D). While mean torque varied smoothly across deciles, there were changes in shape of the wingstroke, including phase shifts. These were stronger in some animals than in others (Fig 2D). In animal J (Fig 2C), the amplitude of the torque around ventral wingstroke reversal (~25 ms) varied and a secondary peak arose in the middle of the upstroke (~37 ms). In animal L (Fig 2D), a similar double peak formed between 50 and 80% of the wingstroke cycle and there was a prominent phase shift. These patterns were consistent for both phase and spike-triggered ensembles.

### Dimensionality reduction identifies two significant features

**Fig 3:**
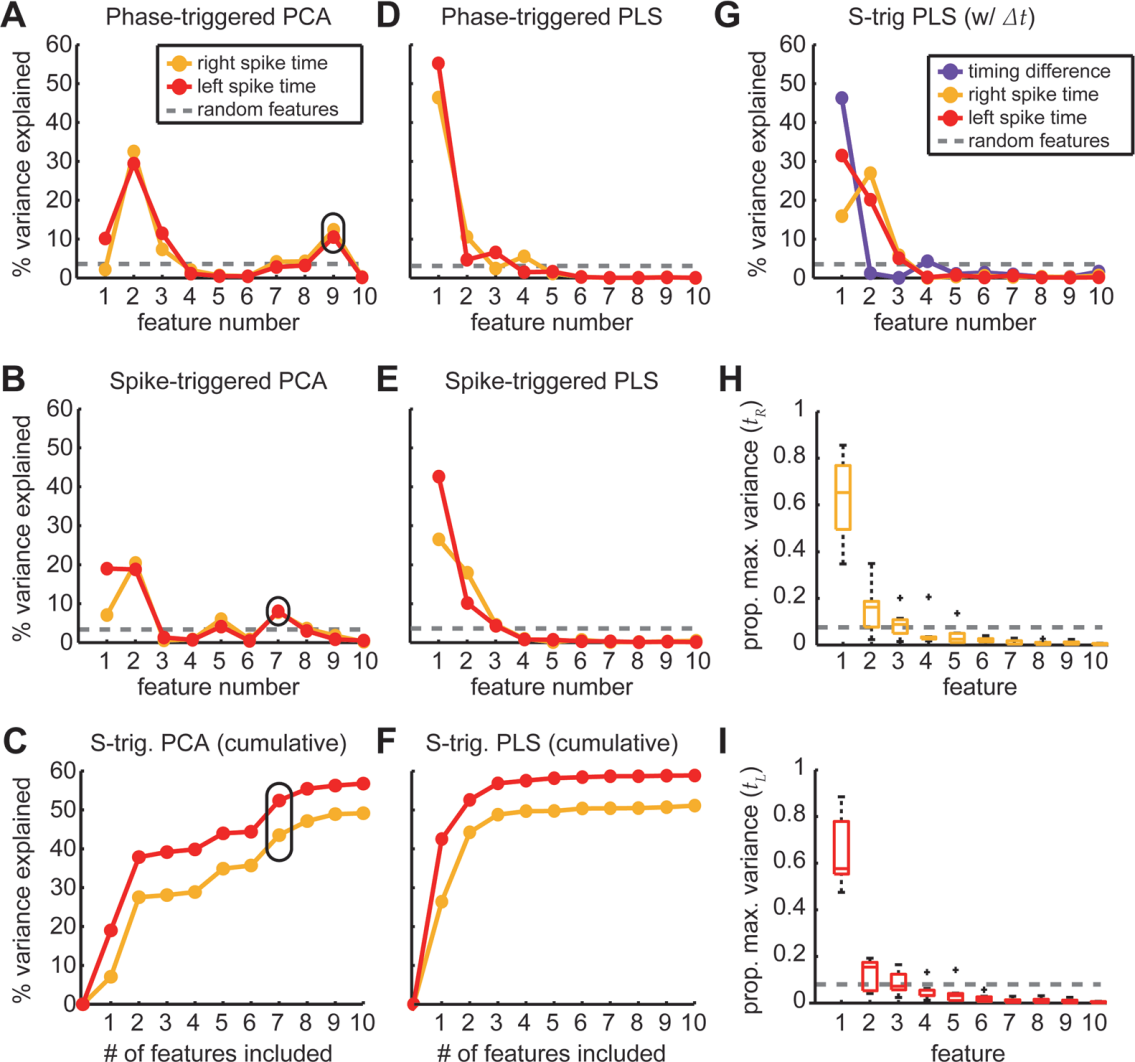
PLS features improve variance explained in motor commands. We extracted 10 features for each dimensionality reduction and alignment method (PCA: A-C; PLS: D-F). We plotted the variance in each muscle timing variable (colored lines) explained by each feature from the phase- (A, B) and spike-triggered ensembles (B, E) as well as the cumulative variance for the spike-triggered case (C, F). To determine the variance explained by random features (dashed lines) we resampled the torque waveforms and muscle timings 1000 times. We used the 99.5% quantile as a threshold for how much variation could be explained by chance from a single feature. Some higher-ranked features in the PCA analyses had significant contributions (circled) above chance. We repeated the spike-triggered PLS analysis with Δ*t* included in the ***U*** matrix to ensure that statistical bias did not change the number of important features (G). To compare across all animals (H, I), we normalized the variance explained by the cumulative variance explained when including 10 features. The box plots indicate the proportion of this maximum explanatory power described by each successive feature (N = 7 animals; 298–620 wingstrokes per animal).

Our next goal was to determine the dimensions along which the torque signal covaried with the motor signals and which of the four combinations of alignment (phase- vs. spike-triggering) and dimensionality method (PCA vs. PLS) best captured this variation. In PCA, the spike-triggered waveforms required fewer features to reach the same explanatory power as the phase-triggered waveforms (Fig 3A and 3B). This is presumably because the spike-triggered ensemble contains more implicit information about the timing variables. PCA features are ranked in order of their ability to describe variation in the motor output. This ordering does not correspond to each feature’s ability to predict motor timing variables: some higher-ranked PCA features explained more motor signal variation than lower-ranked features. Some lower-rank PCA features also describe variability in the waveform ensemble that is not correlated with spike timing. As a result, the cumulative sum of the variance explained by the PCA features did not have a constant plateau (Fig 3C).

Ranking features based on how well movement, ***M***, explains muscle activity, ***U***, is exactly the strength of PLS and, as expected, the PLS features are ordered such that they explain successively more of the variance in the motor timing signals (Fig 3D–3F). The spike- and phase-triggered alignments performed comparably under PLS decomposition (Fig 3D and 3E) presumably because the spike timing information is now incorporated in the dimensionality reduction. However, with spike triggering, there was smoother accumulation of variance concentrated in the first features (Fig 3F). Including Δ*t* along with *t_L_* and *t_R_* in the original ***U*** matrix did not affect the number of motor features extracted from torque (Fig 3G).

The cumulative variance explained in timing variables does not reach 100% because features iteratively maximize the cross-covariance; the procedure aims to capture variance explained by the timing variables, not all variance in the output. The amount of variance explained also differs across animals. To combine all individuals, we scaled variation explained to the variance captured with 10 features (Fig 3H and 3I). We did the same for the random features extracted from resampling the torque for each animal. The first two features explain significantly more variation than chance in the timing of both the left and the right muscle. While the variance generally drops off rapidly after the first feature, the second feature was important in some animals and so we retain it. Our conclusions about synergies do not depend on the inclusion of the second feature.

**Fig 4:**
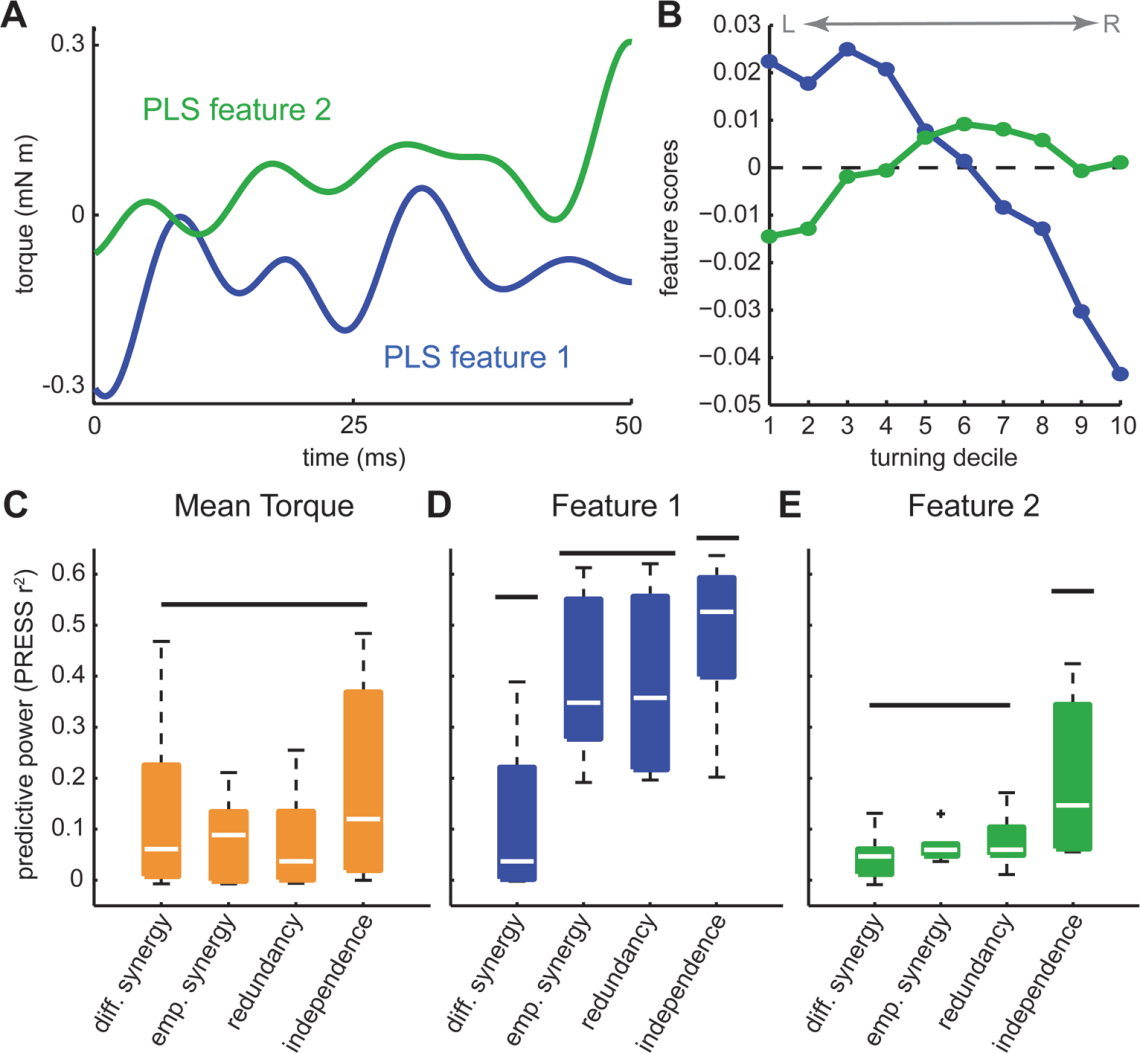
Identified features support the independence model. We plotted the timecourse (loadings) of each significant PLS feature (A). The decile-averaged score for each feature varied from one extreme of turning to the other (B). We determined how well different subsets of the timing variables (the independence, synergy, and redundancy models) predicted either the mean torque (C) or the feature scores (D, E) with a hold-one-out PRESS statistic. The box plots represent the mean and quartiles, with whiskers encompassing all non-outliers. Horizontal lines indicate statistically separate groups.

In each animal analyzed, the two PLS motor features form a low dimensional basis describing the torque or movement matrix ***M*** and vary with left-right turning. Features generally had four periods of oscillation per wingstroke, consistent with the mean torque waveform, or spike-triggered average (“STA”; Fig 4A). The score of the first feature correlated with the degree of turning from left- to rightmost. The second feature’s score had a maximum at intermediate torque values, corresponding to straight flight, and decreased during extreme left and right turns (Fig 4B).

The shapes of the extracted features make sense given our understanding of the control of flight. Varying the amplitude of the first feature has the greatest effect on torque at the beginning and midpoint of the wingstroke (Fig 4A). These points correspond to ventral and dorsal wingstroke reversals, critical moments for flight control [44]. Furthermore, the features oscillate at approximately four times the wingstroke frequency, which is consistent with the patterns observed in the aerodynamics of a robotic *Drosophila* wing model [45].

### PLS motor features reject synergy models

Having identified PLS features that capture the variation in torque relating to variation in the motor signals, we now use this feature basis to test for synergies (Fig 1G). We compared the variation in the torque captured by reduced combinations of the motor signals (the synergy or redundancy models) with that predicted by the two signals together (the independence model). We predicted mean torque and the identified torque features through regression and cross-validation (PRESS). When we predicted only the mean torque, but not the torque features, the one-variable synergy models performed as well as the two-variable independence models (p > 0.1; Fig 4C), supporting the interpretation that the two muscle commands act as a synergy. However, when we considered either of the two PLS features, we found that the independence model significantly outperformed the redundancy or synergy models (p < 0.05 in all cases; Fig 4D and 4E). This was true even for the first feature alone, which indicates that our conclusions do not rely on the iterated extraction of features—even the first feature is encoded in both *t_L_* and *t_R_*.

### Torque reconstruction improves with independently driven motor features

**Fig 5:**
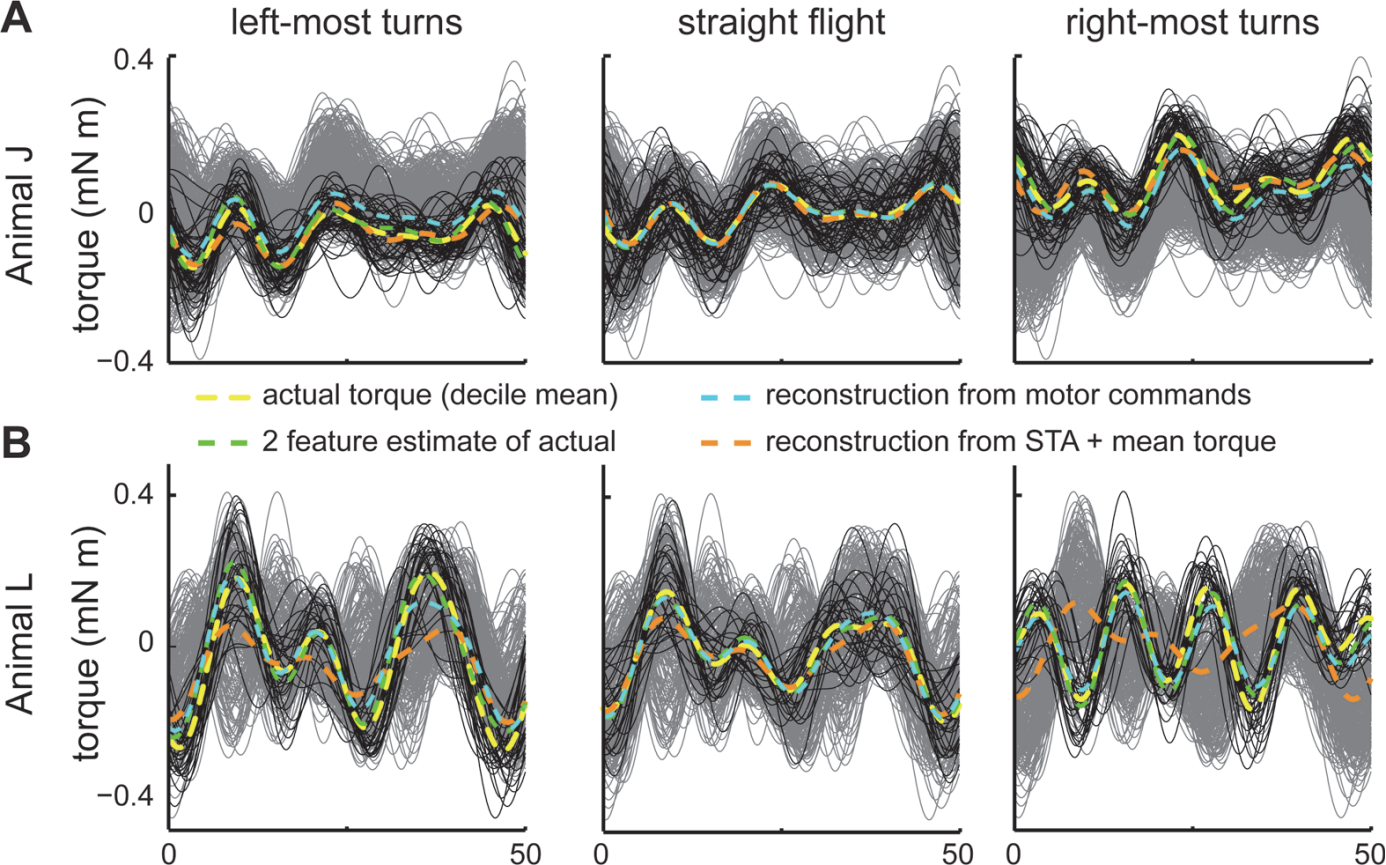
Reconstructing the torque waveforms. Different reconstructions of the decile-averaged torque waveforms are shown for animal J (A) and animal L (B). The full ensemble is in grey and the decile ensemble in black. The measured torque (yellow) was compared to the reconstructions based on: the two feature projection of the measured torque (green), predicting the torque from subsets of the motor signals via the features (cyan), or by predicting the mean torque alone and adding it as an offset to the STA (orange).

**Fig 6:**
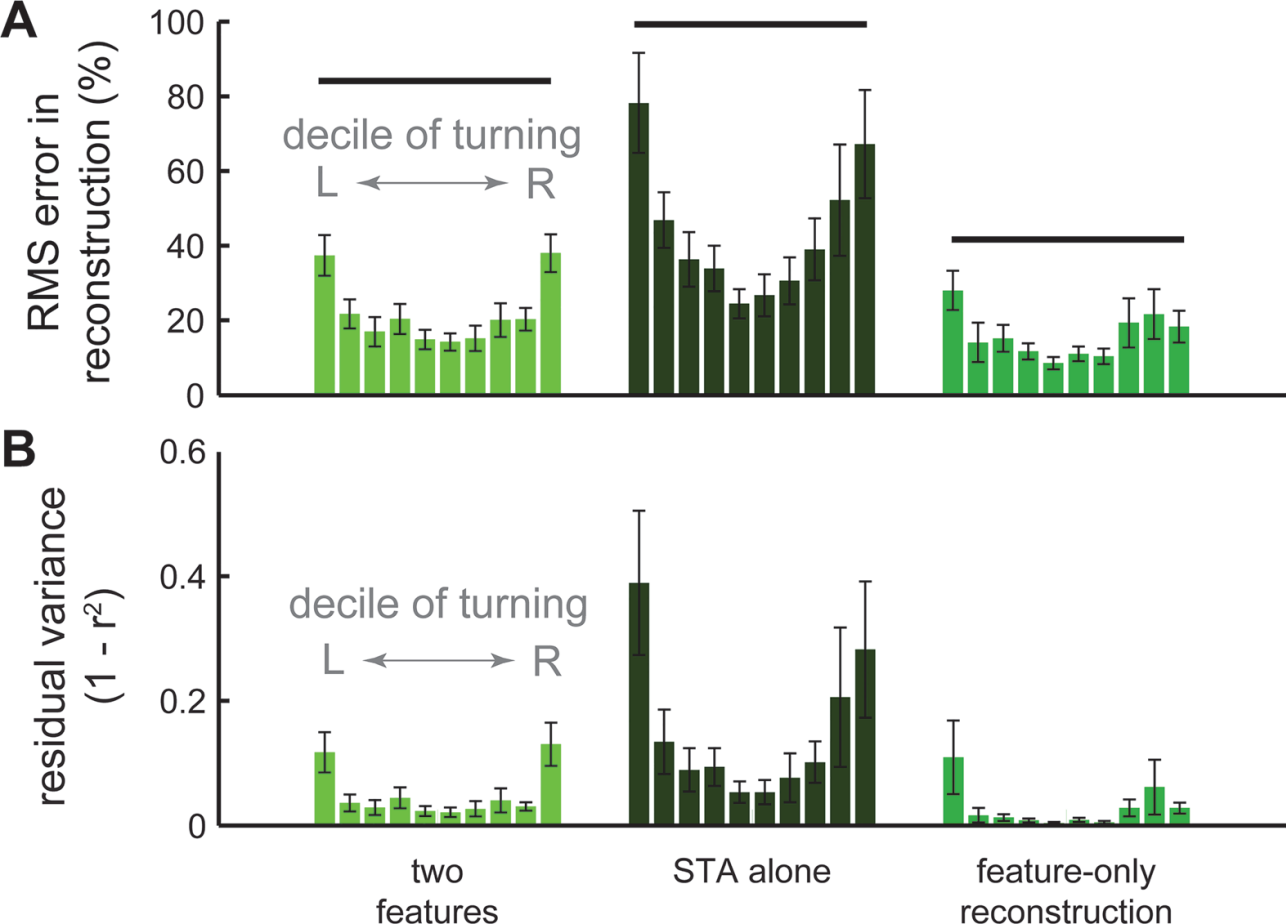
STA and features reconstruction performance. Reconstruction error for each decile of wingstrokes averaged across all animals was quantified for the STA and two-feature projections of the torque data using the normalized RMS error (A) and the unexplained, or residual, variance (B). The STA-alone reconstruction uses the spike-triggered average for each individual moth as the basis for comparison. The feature-only reconstruction compares the torque waveform constructed from the motor signals to the two feature projection of the measured waveform (rather than the full measured waveform). This demonstrates how well the motor signals can predict the features themselves rather than the entire within-wingstroke torque. Horizontal lines above each group of bars in (A) indicate statistically separate groups.

**Fig 7:**
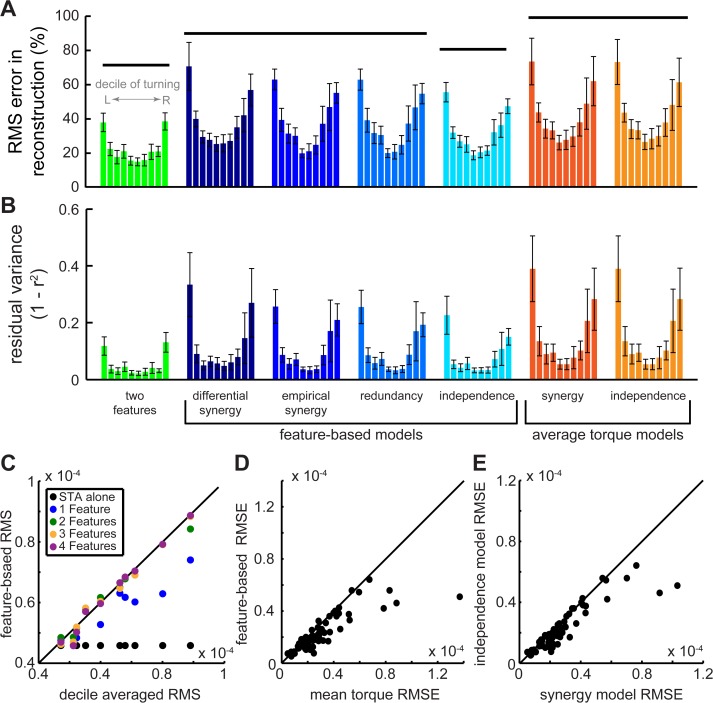
Comparing feature- and mean torque-based reconstructions. We compared reconstruction performance with normalized RMS (A) and unexplained variance plots (B; N = 7 animals) as in Fig 6. Low error values indicate better performance. Horizontal lines indicate statistically separate groups. Significant differences exist between the deciles within each distribution (repeated measures ANOVA, P < 0.0001). The RMS of the reconstructed torque waveform with a variable number of features (0–4) is also plotted against the RMS of the measured torque waveform (C) for all the deciles of animal L. Deviation from unity (solid black line) shows the error in the RMS reconstruction. To compare the feature-based independence model to the mean torque (D) and the synergy models (E), we plotted the RMS error of each against the other for all deciles and all animals (N = 70 animal-deciles). Points below the unity line indicate improved performance (smaller error) with the feature-based independence model.

Rather than just predicting the feature scores, we next addressed how well these descriptions of the motor commands and PLS features could reconstruct the entire torque waveform. The first two PLS features produced reconstructions of torque that closely matched the decile means (Figs 5A, 5B and 6A), describing 95% of the variance and 70% of the residual variance after correlating the STA to the waveforms (Fig 6B). If we predicted the amount of each feature (its score) from the motor signals, the reconstruction was necessarily worse than if we used the measured feature scores (P < 0.001; Figs 5, 7A); compare cyan and green), but the resulting reconstructions were still significantly better than those based on mean torque alone (P < 0.003; Fig 7A, 7B and 7D) or using the STA without any added features (P < 0.0004; Fig 6).

The PLS feature-based analysis improved reconstruction primarily by matching the shape and phase of the torque waveform (Fig 5A and 5B). The independence model accurately reconstructed 91% of the average torque waveform across all turning deciles (Fig 7B) and 53% of the residual variance after accounting for the STA. Wingstrokes during more extreme turns were less completely reconstructed (Fig 7A and 7B), but this is not surprising given that there are other muscles involved in turning whose activity we did not record. If we restrict the reconstruction to the torque projected into the two-feature subspace, the motor signals are even more accurate and consistent across deciles (Fig 5).

Using the RMSE waveforms for all the deciles, we first confirmed that adding more features beyond the first two did not improve reconstruction (Fig 7C). Reconstruction based on the independence model (using both *t_R_* and *t_L_* to predict the feature scores in Equation 16) outperformed both synergy models and the redundancy model in minimizing the RMS error in the torque—even when controlling for the differences across deciles (effect of model in two-factor ANOVA with decile; P < 0.05 in all cases; Fig 7A and 7D). As before, we only rejected the synergy models when considering PLS features because when considering the mean torque alone, the independence and synergy models were equivalent (two-factor ANOVA; P > 0.1; Fig 7A and 7E).

To summarize reconstruction performance across all deciles into a single performance measure, we took the mean ratio between the decile RMSE of each model and the RMSE of the two-feature reconstruction, which is the best two-dimensional reconstruction possible:

$$error\;ratio=<RMSE_{model}/RMSE_{2F}>.$$ (18)

**Fig 8:**
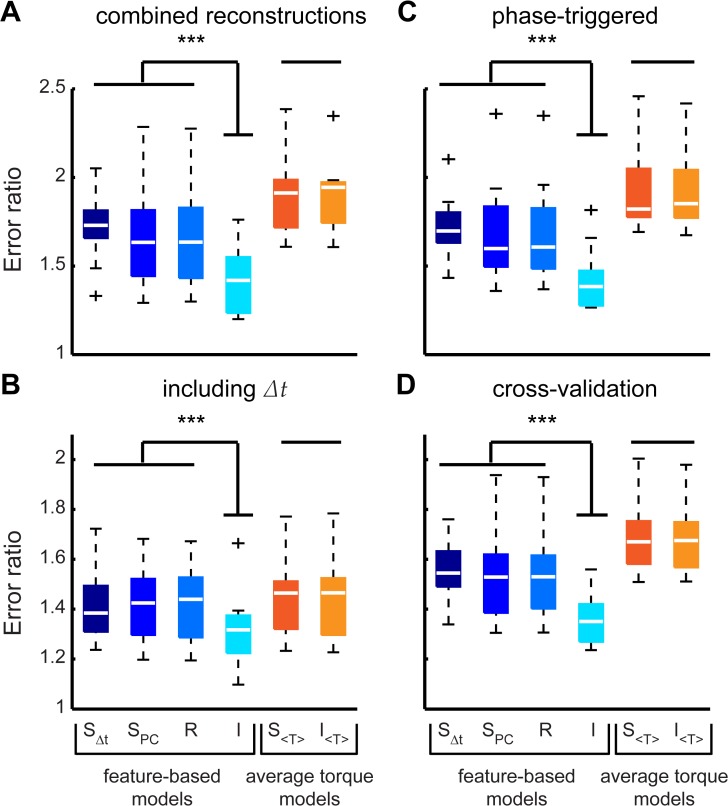
Cross-validation and sensitivity. To combine reconstruction performance into a single error metric we normalized the RMS of the error for each decile (averaged across animals) to the RMSE of the two feature reconstruction (*** indicates P<0.001 for all paired conditions). In addition to the standard reconstruction (A) we repeated the feature extraction and model testing with *Δt* included in the original U matrix (B) and with the phase-triggered rather than spike-triggered torque waveforms (C). Finally, we cross-validated (D) the reconstructions performing 1000 replicates with 70% of the wingstrokes as a training set and 30% withheld for a test set. Model abbreviations: 2F—two features, S_Δt_—differential synergy, S_PC_—empirical (PCA) synergy, R—redundancy, I—independence, S_< *τ* >_ mean torque differential synergy, I_< *τ* >_—mean torque independence.

This allows for paired tests across all animals and wingstrokes (Fig 8A; paired t-tests comparing feature-based synergy models to independence P < 0.001 in all cases; average-torque based: P = 0.94). The conclusions held even if the most extreme left and right turning deciles (20% of the data) were excluded from the analysis (feature based: P < 0.003; average torque based: P = 0.7). To test if the rejection of the synergy hypothesis was due to statistical bias introduced by not including a linear combination of *t_R_* and *t_L_* in the original ***U*** matrix of motor signals, we repeated the entire analysis including Δ*t* in ***U***. The independence model still outperformed synergy models in the feature based cases (Fig 8B; feature based: P < 0.0004; average torque based: P = 0.98).

One concern about using the spike-triggered ensemble is that variation in torque may itself be phase locked. In that case, spike triggering could introduce a spike-timing dependent signal that might bias our data. It is unlikely that variation due to changes in the muscle’s timing would be phase locked independent of muscle timing, but to check for this bias we repeated the feature extraction and reconstruction using the phase-triggered torque waveforms rather than spike-triggered ensembles. The results were the same as before (Fig 8C; feature based: P < 0.001; average torque based: P = 0.94).

**Fig 9:**
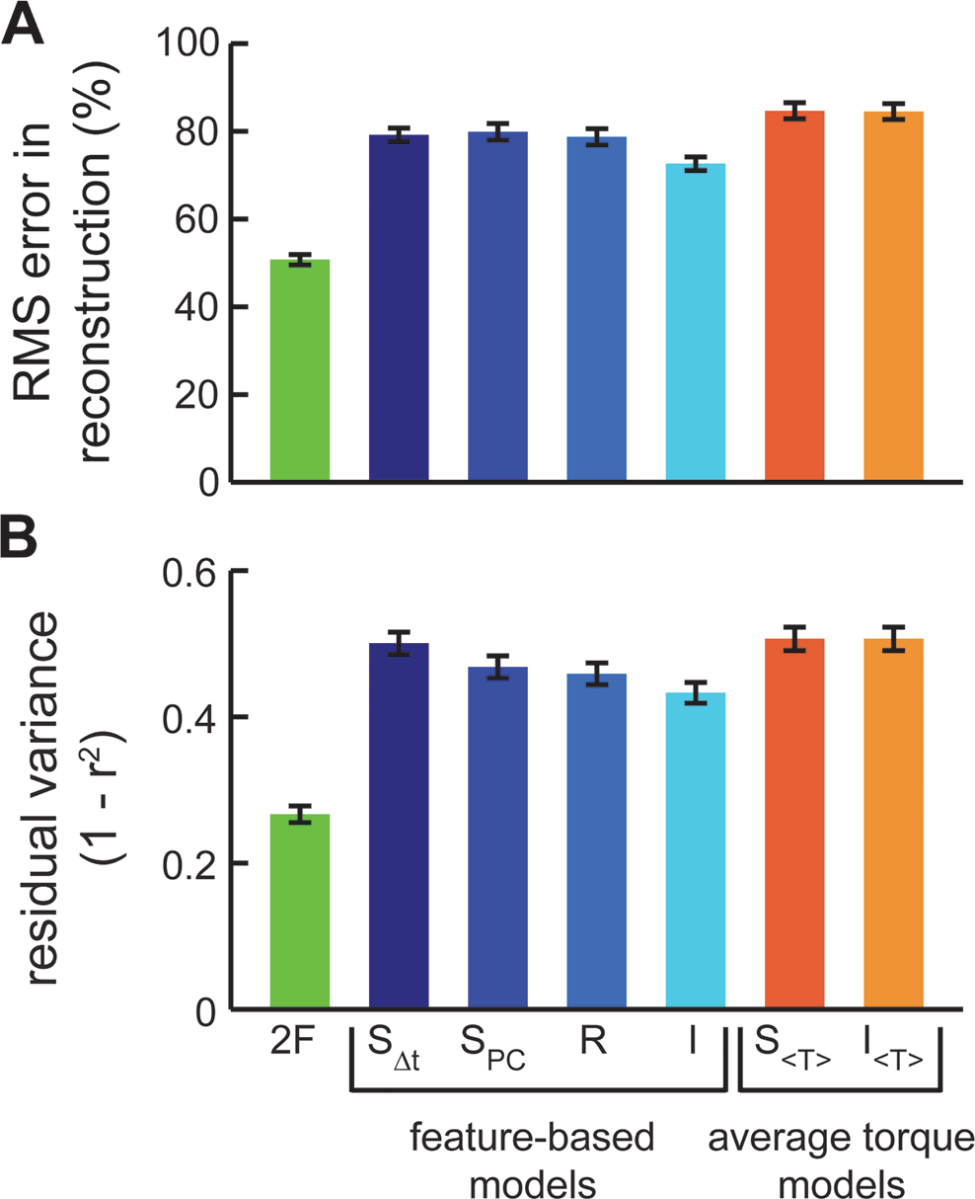
Reconstructing individual wingstrokes. RMS error (A) and residual variance (B) measures of the average performance for reconstructing individual wingstrokes show necessarily greater error, but consistent patterns across synergy and independence models. Colors and models correspond to those of Fig 7. Data are from reconstructing all of the individual wingstrokes from animal L (n = 298 wingstrokes) with error bars being s.e.m.

Finally we challenged the models to be more predictive. First, we tested that the results were robust to cross-validation (Fig 8D). Second, we reconstructed the torque of individual wingstrokes rather than decile averages. RMS errors were naturally higher (Fig 9A), but the reconstructed waveforms were highly correlated with the measured torque (Fig 9B) and the synergy models were still rejected in feature-based analyses (paired t-test compared to independence model; all P < 10^−7^).

## Discussion

### PLS features support independent rather than synergy encoding in the DLMs

Only two out of 500 possible motor features were required to describe the variation in torque encoded in changes in DLM activation during visually-induced turning (Fig 3D). Reconstruction of decile averages (Fig 7C), cross-validated predictions (Fig 8D) and individual wingstrokes (Fig 9) are all improved by incorporating PLS features. We found that retaining the timing of both DLM activations captured more of the variance in torque than compressing these timing variables into a single linear combination, or synergy (Fig 4D and 4E). The independence model also more accurately decodes within-wingstroke torque by reconstruction (Figs 7D, 7E and 8A).

The need to retain independent variation in the DLMs' activation was only revealed using the PLS feature basis. The synergy models are sufficient to account for the torque averaged over the wingstroke (Figs 4C, 7D and 8). When considering turning torque only from wingstroke to wingstroke, the difference in timing (the differential synergy) may be sufficient to describe dynamics. However, independent variation in the two muscles is necessary to encode within-wingstroke dynamics. This does not imply that each muscle’s activation is orthogonal. These within-wingstroke dynamics are likely critical to flight control. A recent Floquet analysis treating the periodic dynamics of flapping flight rather than just the wingstroke-averaged forces demonstrated that ignoring within-wingstroke dynamics alters the stable and unstable modes of moth flight [46].

The advantages of the PLS-based approach come in dealing with spike-resolved motor signals and in testing synergies in terms of how well motor variation is encoded rather than how much variation in muscle activation is described. Most synergy analyses consider a large number of muscles, each described by a single, time-varying activation variable [9,10,19]. This study connects individual spike-level encoding of movement with muscle synergy questions. Additionally, while it is common to identify the force vectors or movements that correspond to muscle synergies [19,47], the extraction of synergies is usually an independent dimensionality reduction. Considering only the variation in muscle activation does not address whether or not this variation is relevant for movement generation. Here, we decompose a high-dimensional representation of torque to identify a relevant basis dependent on the variation in the muscles’ activity. We assess synergy performance against this basis.

Whether muscle synergies represent a general strategy to simplify control remains an open question [10,20]. We found that a muscle representation that retains the independent timing of both muscle activations captures more behavioral variation in torque output than three specific synergy alternatives [14]. If recordings from more muscles were included, more complex synergies might exist. For example, some of the information encoded in the timing of the left DLM might also be shared with a steering muscle’s activity. This is always a concern with encoding studies, which alone do not establish causality. Fortunately, in this case we do know that the DLMs are causally involved in producing turning torque [14]. More importantly, while we cannot say if a larger set of recordings might not express synergies, we do know that the variation in the downstroke muscles must contribute to at least two separate synergies—they cannot be expressed in a single linear dimension even with more muscles included.

For the present analysis, we constructed synergies from linear combinations of the motor signals, ***U***, as a separate step from the PLS feature identification to be consistent with prior synergy analyses [9,32]. However, PLS provides an alternative way of constructing synergies. We obtain not only the motor features, ***P***, but also a matrix of equal dimension ***Q***, whose vectors, ***q_i_***, represent a non-orthogonal set of synergies for the motor signals, ***U*** (Table 1, Equation 5). This approach scales well as dimensionality increases and could also be advantageous for analyzing population encoding of complex sensory stimuli. In our case, reanalysis using the first vector ***q_1_*** instead of the first PC of the DLMs’ activations does not change our conclusions.

It is important to note that these methods are linear, and that our conclusions about the nature of motor encoding are based on the quality of linear correlation. It is possible that nonlinear analyses that take into account higher-order correlations may reach different conclusions. For example, information-based methods provide an alternative to covariance approaches that satisfy some of these same goals. The technique of maximally informative dimensions [48] seeks a feature basis which captures the most mutual information between input and output, where the output typically is the occurrence of a single spike [49]. This approach can likely be generalized to discover complex output symbols that preserve mutual information [50]. Such techniques can handle naturalistic stimuli and minimize *a priori* assumptions about the statistical structure of the data, but they typically require estimating the marginal (or conditional) probability distribution of the outputs with respect to the inputs. This is very challenging when the dimensionality of both the input and output becomes large (although see [51]). In contrast, PLS readily scales to a large number of inputs and outputs because it relies only on estimating the cross-covariance structure in the data.

### Motor features in the context of insect flight

That DLMs encode discernible torque variation is itself surprising because these muscles were long thought not to have any significant role in control [13]. DLMs can cause significant turning variation because small changes in their timing produce large changes in power [14]. However, other muscles almost certainly play a role in turning as well. In particular, small steering muscles modulate wingstroke angle (e.g. the 3rd axillary muscle) and demonstrate correlated phases of activation [13,30,52]. The role of steering muscles is one reason why the PLS approach is critical. PLS extracts features from the movement matrix, ***M***, only if they covary with motor signals, ***U***, and should ignore variation that is due to steering muscles but not encoded in the DLMs. Therefore, while a mechanistic understanding of turning control is not yet complete, our results do indicate that each DLM independently encodes information about the optomotor response.

One reason why synergies may be present in nervous systems is to allow task control with a smaller set of command variables [9,10]. Optomotor sensory input is bilateral, at least in flies, because each eye possesses distinct populations of left and right directionally selective cells [53]. However, this information could be centralized into a single descending command setting the timing difference between the flight muscles. The fact that we reject a reduced representation for the pair of DLMs indicates that visual information can separately modify the descending commands to each of the two DLMs, in addition to its known feedback to steering muscles [13].

### PLS feature analysis captures task relevant variation when multiple neuromuscular signals encode movement

A PLS dimensionality reduction of torque incorporates multiple, continuous variables to describe the motor signals, while PCA can only incorporate information about the timing of one spiking event (or pattern of spikes), via spike triggering [5]. By ranking features based on the cross-covariance, the PLS approach only captures motor variation relevant to the changing motor signals, while to some extent excluding components that are unrelated [12,35]. These factors account for the improved performance of PLS compared to PCA (Fig 3) and should only improve further as the dimensionality of both the motor signals and of movement increases. Our analysis considering only two muscles is the minimal case in which PLS could outperform a spike-triggered PCA. Even in this case, the abilities of PLS to deal with the challenges of multiple high dimensional datasets and incomplete representation are necessary to discover independent encoding.

Overall, PLS-based feature analysis is a data-efficient method to extract a reduced representation of a multiple input-multiple output (MIMO) data set. Here, the approach improves predictability of wingstroke variability and our understanding of the motor program. In contrast to encoding in the central nervous system [54–56] and sensory systems, there have been few applications of dimensionality reduction approaches to encoding in the peripheral motor system. Just as sensory encoding reveals the patterns of stimuli to which neurons respond, the encoding of movement in the activation of multiple muscles reveals the structure of the motor program. New approaches like PLS-based feature analysis set the stage for understanding how the peripheral nervous system represents locomotor control.
